# Bone mineral density in people living with HIV: a narrative review of the literature

**DOI:** 10.1186/s12981-017-0162-y

**Published:** 2017-07-26

**Authors:** M. J. Kruger, T. A. Nell

**Affiliations:** Department of Physiological Sciences, Stellenbosch University Main Campus, Stellenbosch, 7602 Western Cape Province Republic of South Africa

**Keywords:** HIV, Body composition, Bone mineral density, QUS, DEXA

## Abstract

Bone health status is largely absent in South Africa, the main reasons being the absence and cost-effectiveness of specific screening equipment for assessing bone mineral density (BMD). Various risk factors seem to play a role, some of which can be modified to change bone health status. Urbanisation is also a public health concern. Changing nutritional, as well as social behaviour, play integral roles in the prevalence and incidence of decreased BMD. Furthermore, human immunodeficiency virus (HIV) specifically, has a negative impact on BMD and although highly active antiretroviral therapy increases the prognosis for HIV-infected individuals, BMD still seem to decrease further. Dual energy X-ray absorptiometry is considered the gold standard for BMD assessment; however, recent developments have provided more cost-effective screening methods, among which heel quantitative ultrasound appears to be the most widely used in resource limited countries such as South Africa.

## Background

**Fig. 1:**
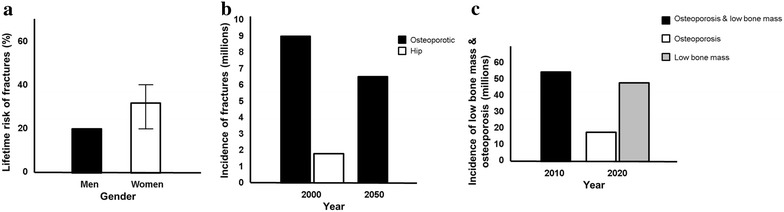
Representative data indicating (**a**) the lifetime risk of fractures, (**b**) the incidence of fractures and (**c**) the incidence of low bone mass and osteoporosis

Osteoporosis, the most common metabolic bone disease, affects almost a quarter of all postmenopausal Caucasian women, the lifetime risk of fracture in these women being 30–40% compared to 20% in men (Fig. 1a) [1]. Worldwide, this risk can vary up to tenfold, depending on the country of origin—the greatest risk reported for the United States of America (USA) [2]. Global statistics regarding the prevalence of osteoporosis, bone mineral density (BMD) and fracture risk are readily available for international countries due to the continuous access to expensive equipment that can screen for bone mass.

The World Health Organization (WHO) developed the Fracture Risk Assessment Tool (FRAX) in order to determine a 10-year probability of the potential risk for future hip fractures or major osteoporotic fractures in women and men between the ages of 40 and 90 years, by using different risk factors related to BMD [3]. In 2000, the incidence of osteoporotic fractures was estimated to be nine million worldwide, with hip fractures contributing 1.6 million (Fig. 1b) [4]. In 2010, projections for the USA (according to FRAX) indicated that approximately 52.4 million individuals older than the age of 50 years would develop low bone mass and osteoporosis [5]. Furthermore, it was predicted that approximately 14 million individuals would be diagnosed with osteoporosis, whereas 47 million will be more likely to have low bone mass in 2020 (Fig. 1c). Since osteoporosis is the most common cause for fractures, it accounts for approximately 1.5 million fractures each year—hip fractures is projected to increase to 6.3 million by 2050, while osteoporosis is projected to increase exponentially due to the aging of the population (Fig. 1b) [6]. Fragility fractures, fractures that occur as a result of a fall from a standing height, lower, or from no trauma at all, can also impact FRAX scoring, although this is poorly understood.

According to the clinical guideline published by the National Osteoporosis Foundation of South Africa (NOFSA) in 2010, the incidence of osteoporosis in white, Asian and mixed race populations in South Africa (SA) seems to be similar to that observed in developed countries, although no accurate fracture data exists for SA [1]. Osteoporosis appears to be less prevalent in SA black populations, similar to data for the USA, although some studies show contradictory results [7–9]. The reasoning behind this being the contribution of fat mass, more specifically visceral adipose tissue (VAT), compared to skeletal muscle mass. What is somewhat concerning is that according to a recent extended report by Sanchez-Riera et al. on the global burden attributable to low BMD as measured by dual energy X-ray absorptiometry (DEXA), no data on BMD at the femoral neck is available for sub Saharan Africa (East and Central), whereas only two studies have reported on BMD in West sub Saharan Africa, and one in South sub Saharan Africa [10].

## Bone mineral density (BMD)

### Definition, classification and guidelines

Bone mineral density (or bone density), a quantitative measurement of bone mass referring to the amount of mineral matter (calcium) in grams per square centimetre (cm^2^) of bones [11, 12], is used in clinical medicine as a surrogate indicator of osteoporosis and fracture risk. According to the National Institutes of Health Consensus Development Panel on Osteoporosis Prevention, Diagnosis, and Therapy, osteoporosis can be defined as a skeletal disorder characterized by increased fracture risk and low bone strength [13].

Several guidelines for the diagnosis and management of osteoporosis have been proposed in 1994 by the WHO. Hough et al. summarized these guidelines into one document for NOFSA [14]. According to the WHO, a subject’s BMD is expressed in relation to a young adult reference mean for female Caucasians (T-score) and grouped accordingly into either normal, low bone mass or osteopenia, osteoporotic, and severe osteoporotic categories [14].

The WHO’s diagnostic criteria for osteoporosis is a BMD measurement equal to or more than 2.5 standard deviations (SD) below the young female (aged 20–29 years) reference mean (T-score ≤−2.5 SD), whereas a patient is diagnosed with osteopenia if they have a T-score between −1.0 and −2.5, and as normal if they have a T-score between 1.0 and −0.9 [14].

According to the WHO, T-scores should be reserved for diagnostic use in postmenopausal women and men aged 50 years or more only [14]. For all other populations, Z-scores should be used instead, although categorising individuals as either osteopenia or osteoporotic should still be acceptable for the femoral neck. The Z-score, calculated in the same manner as the T-score, compares a person’s bone density to that of an age-, sex-, and race-matched population. This scoring system is usually reserved for youngsters and adolescents; however, considering that these individuals are still growing, the Z-score should also account for pubertal stage and body size. Specialists should also be cautious when diagnosing osteoporosis (a Z-score of ≤−2.0) in children with this scoring system, as one also needs to consider the presence of at least one fragility fracture [15]. Seeing that the Z-score is age-matched, it can also point to a secondary cause of osteoporosis, other than age in older individuals [16].

### Physiology and pathophysiology

**Fig. 2:**
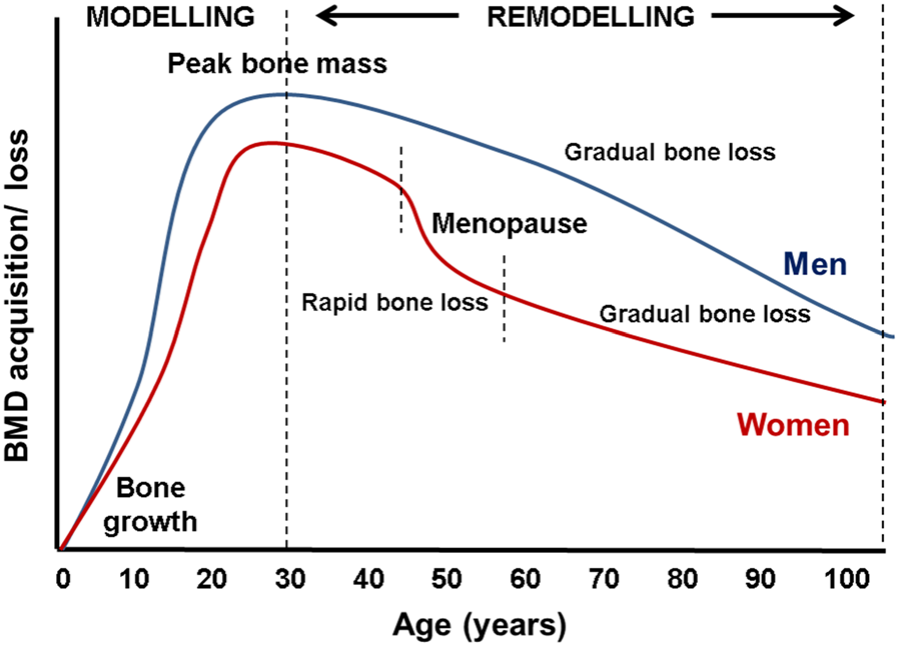
The general pattern of bone development and loss over time. During years 0–20, bone starts to grow, during the ages of 20–30 years, modelling takes place and peak bone growth is reached. This is followed by the remodelling phase where bone loss is evident

In order to fully comprehend the debilitating effects of osteoporosis, a short summary of the natural life history of BMD acquisition and loss is included. Normally, during childhood and the teenage years, bone density accumulates and bone grows in both size and strength (*growth phase*), as new bone is added to the skeleton faster than old bone is removed or resorbed (Fig. 2) [17]. After the growth spurt stops, bone formation continues faster than resorption until *peak bone mass* is reached at about age 25–30 [18, 19]. Bone density is then maintained for about 10 years, at which point bone mass may remain relatively stable (*remodelling phase*), whereafter the age of 35, both men and women will gradually lose 0.3–0.5% of their bone density per year as part of the aging process [20, 21]. Between the ages of 45 and 55, the menstrual cycle in women ceases, at which point women start to loose bone rapidly in the first 4–8 years thereafter, as a result of decreased oestrogen production [22]. By age 65, men and women tend to lose bone tissue at the same rate, which is much more gradual and continues throughout life.

Considering normal bone growth, osteoporosis is thought of as a silent disease, since bone loss usually occurs without any signs or symptoms. It is also characterized by low BMD, increased bone turnover and deterioration of bone microarchitecture [23]. Although BMD is used in the diagnosis of osteoporosis, low BMD is not the only risk factor for fractures and is not often used on its own to identify fracture risk [24].

Various risk factors are associated with the development of osteoporosis. These include unmodifiable risk factors such as age (the older, the more at risk), sex (females are more at risk), ethnicity (variable results, but pointing towards non-caucasians being more at risk), family history of osteoporosis (immediate family) and prior vertebral fracture or fragility fracture [24–26]. Modifiable risk factors include low body mass index (BMI), particularly in postmenopausal women, exposure to oestrogen, physical activity, long-term glucocorticoid treatment and long-term anticonvulsant therapy (phenytoin, phenobarbital and carbamazephine) [24–26]. Additionally, nutritional status also plays a significant role—impaired absorption or low dietary intake of vitamin D and calcium and excessive use of alcohol (>3 units per day) and tobacco are also risk factors for low BMD [27]. Secondary causes for osteoporosis also exist and include type 1 diabetes, rheumatoid arthritis *osteogenesis imperfect* (brittle bone disease) in adults, *anorexia nervosa*, hyperparathyroidism, untreated long-standing hyperthyroidism, hypogonadism or premature menopause (<45 years), chronic malnutrition or malabsorption (e.g. coeliac disease, inflammatory bowel disease) and chronic liver disease.

Currently, the WHO uses the FRAX index assessment tool to predict fracture risk for a 10 year period in people with low BMD by incorporating various clinical risk factors in its calculation [28]. These include age, sex, race, height, weight, body mass index, fracture history of the patient and parent, current smoking, glucocorticoid usage, the presence of rheumatoid arthritis, secondary causes of osteoporosis, alcohol intake and the BMD measure of the femoral neck. It has been suggested that FRAX only be used to calculate fracture risk for patients who have T-scores between −1.0 and −2.5 in the spine, femoral neck, or total hip region, but not when patients are already on pharmacologic treatment for osteoporosis. According to NOFSA, treatment should also only be considered when the 10-year risk of major-osteoporotic-related fractures as calculated by FRAX are ≥20% [29, 30]. Although FRAX includes a vast number of risk factors, it neglects other important factors, such as 25-hydroxyvitamin D [25(OH)D], physical activity, risk of falls, and biochemical bone markers. Furthermore, multiple risk factors could account for the presence of secondary osteoporosis; however, the risk score does not distinguish between any of these risk factors, which could impact the predicted risk differently.

Different methods exist to assess BMD. These methods will be discussed in the next section, with specific focus on the advantages and disadvantages of each.

### Bone mineral density assessment methods

#### Dual energy X-ray absorptiometry (DEXA)

The use of DEXA in the diagnosis and management of osteoporosis is considered the gold standard to determine loss of BMD [31]. Despite being considered the best clinical tool for diagnosing osteoporosis, DEXA does have some limitations. The biggest critique is that it is influenced by bone size, which suggests that the larger the bone, the greater the BMD, irrespective of its volume. This poses a potential problem since men display higher BMD, even though they might have similar or lower volumetric bone mineral density (vBMD) [32]. Furthermore, two-dimensional (2D) DEXA can also be influenced by artefacts such as aortic calcification and degenerative changes in the spine [23]. Nonetheless, and even though a skilled operator is necessary for its interpretation, DEXA is known for its speed, low radiation dose, moderate cost and ease of operation [33]. In the SA setting specifically, DEXA is not readily available in all clinics; therefore, various other diagnostic assessment tools are implemented to determine BMD. Although BMD can be assessed with other imaging tools, the DEXA T-score must be used as reference standard to compare and validate other BMD modalities.

#### Quantitative computed tomography (QCT)

In contrast to DEXA which provides 2D scans of bone, quantitative computed tomography (QCT) provides three-dimensional (3D) measures of vBMD with the potential to examine specific compartments within the body separately, such as trabecular and cortical bone, as well as provides a better estimate of the bones’ cross-sectional geometry (bone shape and size) [34, 35]. This technique is usually used in conjunction with DEXA for better prediction of fracture risk and osteoporosis, as QCT is predominantly perceived as a research tool [34].

Volumetric QCT is regarded as a quicker and technically less demanding acquisition tool, even though skill is required for its interpretation, and is more widely available since a whole body scanner can be used for image analysis with high precision. However, the individual being scanned is exposed to modest radiation, and depending on the resolution, an increased radiation dose is often required. Results from the study by Pickhardt et al. suggest that both QCT and simple non-angled region-of-interest (ROI) attenuation measurements (measures employed in colonography CT, CTC) of the lumbar spine are effective for BMD screening [36]. Data also suggests that single-level ROI vertebral attenuation measurement at CTC is reproducible, requires little effort, and adds no additional radiation or costs, but can provide valuable BMD data for osteoporosis screening [37]. Furthermore, by applying either QCT analysis to non-contrast-enhanced CT studies, individuals at risk for osteoporosis can be identified. However, a DEXA scan is still needed if an individual at risk for osteoporosis is identified, even though it has been suggested that QCT may be better for BMD assessment [38].

#### Magnetic resonance imaging (MRI)

Conventional proton magnetic resonance imaging (MRI) is one of the few non-radiative methods used to characterize bone based on mineral and matrix, and produces a negative image of the bone substance [39]. The microarchitectural details of the trabecular network of the bone is geometrically analysed to determine the volume of the bone substance (similar to BMD) if an image is produced in which one can differentiate between the marrow spaces and the trabeculae of the bone, which is most often a difficult task in human subjects [39]. However, if the image has insufficient dimensional resolution, textural information can be obtained about the trabecular network of the bone which may be related to bone mass [40, 41]. It is this ability of MRI to reflect the micro physiology of tissues that makes it such a powerful tool allowing it to better differentiate patients with and without osteoporotic fractures compared to normal BMD. More specifically, the ability to use MRI for vBMD calculations without losing soft tissue signals makes it more clinically attractive even though using MRI to quantify BMD is somewhat difficult due to the low proton signals in mineral [42].

Several improvements have been made to MRI to overcome the problem of low proton signals. Solid state P31 nuclear magnetic resonance projection MRI have been developed which enhances the phosphorus signals to determine BMD more accurately [42], as well as the more recent development of ultra-short echo MRI [43]. Furthermore, high-resolution MRI has been developed in order to quantify the trabecular architecture at the micro-meter level [44, 45], a difficult process which requires standardization at each stage (high resolution MR images consists of several stages) in order to ensure a high degree of reproducibility [46]. Although various studies have been performed in order to optimize image acquisition, post processing, calibration and validation of measurements using MRI, these methods remain technically challenging if not performed by a trained professional.

#### Quantitative ultrasound (QUS)

Recent developments in densitometry technology have provided alternative methods, among which heel quantitative ultrasound (QUS) appears to be the most widely used. According to the ISCD, calcaneal QUS is the only recognized measurement of QUS to determine bone health status due to the abundance of research done on this particular bone compared to other bone segments [47]. QUS, albeit inexpensive, portable, ionizing-radiation-free and proven to predict hip fractures and all osteoporotic fractures in Caucasian postmenopausal women and elderly men, also requires a trained technician to interpret the results obtained [48, 49]. QUS of the calcaneus has also been shown to accurately predict osteoporotic fractures in elderly female populations [49]. Furthermore, it has the potential to separate osteoporotic individuals from healthy bone individuals [28].

Some of the key features of QUS are its ability to measure the speed of a sound wave (SOS in meter per second—m/s) and its attenuation as it travels through bone. The latter is termed broadband ultrasound attenuation (BUA, dB/mHz), which occurs as a result of the energy that is absorbed by the soft tissue and bone when the sound waves travel through them. It has also been shown that SOS correlates well with BMD [50, 51], whereas BUA is influenced by certain structural characteristics of trabecular bone, such as porosity [50, 52]. A more complex parameter has also been developed from the combination of SOS and BUA, and is used to measure stiffness. This is termed the stiffness index (SI) and has been shown to be more useful in identifying subjects with low BMD and thus high fracture risk [53]. Studies by Gonnelli et al. and Mészáros et al. indicated that both SOS and BUA can be used to distinguish between individuals with and without fractures, however, SOS seem to be somewhat more useful in men [54, 55]. In contrast, according to a study by Ndongo et al., BUA was the most useful measure for identifying factors associated with bone status, such as age, weight and physical activity in urban Sengalese women [56].

Due to the fact that the principles behind QUS and DEXA’s ability to determine bone health differs, the same cut-off points may not be useful for both QUS and DEXA [47], as DEXA’s cut-offs may over/underestimate the true incidence of osteoporosis [57]. Although DEXA is the standard reference method for diagnosing osteoporosis, a recent study in Dakar, Senegal have employed ultrasound to determine BMD and have found it a useful tool for assessing BMD especially in areas where DEXA is unavailable as is often the case in SA [58]. In this particular study, human immunodeficiency virus (HIV)-infected patients have reduced QUS BMD in comparison with subjects from the general population. However, there was no association between QUS BMD and duration of treatment—at least 5 years on highly active antiretroviral therapy (HAART). Since the validated reference method for bone density assessment couldn’t be used, the clinical significance of these results in terms of osteoporosis remains unknown.

It is evident from the studies mentioned above that QUS has the potential to not only predict fracture risk, but also to determine BMD. To complement the results obtained with QUS, specific biochemical markers can be used in conjunction with BMD testing to determine bone health status.

#### Biochemical-specific markers

##### Bone turnover markers

Bone turnover is the main contributor to both quality and quantity of bone. An imbalance between bone resorption and formation leads to a net loss or gain of bone tissue. High bone turnover results in bone loss and abnormal bone microarchitecture, whereas, low bone turnover results in increased bone mass, accumulation of microdamage, and bone fragility [59].

Surrogate markers of fracture risk—bone turnover markers (BTMs) and BMD—can be used to monitor a specific treatment response. High levels of bone BTMs were shown to correlate well with increased fracture risk in postmenopausal women [60]. In various studies regarding therapeutic agents, BMD and BTMs were shown to respond differently, a change in BMD only observed after about 1–2 years [61], whereas changes in urinary levels of BTMs were observed within a couple of months [62, 63]. In a study by Bjarnason et al., short-term changes (3–6 months) in BTM was associated with long-term changes (1–2 years) in BMD, therefore both BTM and BMD can be used, albeit at different times [64].

Although several studies have investigated numerous biochemical markers of bone turnover to determine fracture risk, it was recommended by Vasikaran et al. to use serum carboxy terminal telopeptide of collagen type I (s-CTX) as the standard bone resorption marker and serum procollagen type I N-terminal propeptide (s-PINP) as the standard bone formation marker [65]. Serum CTX is shown to be specifically influenced by renal function, diurnal rhythm and food intake, peaking early in the morning and is at its lowest in the afternoon. Food intake also tends to decrease these levels; therefore, it is imperative that sampling time be in the morning and in a fasted state. Serum PINP on the other hand is not influenced that greatly by diurnal rhythm, food intake or temperature.

##### Biochemical and nutritional markers

Several biochemical markers can also be assessed to determine the contributing factors associated with osteoporosis. These include serum total calcium, albumin (to correct for calcium if data is skewed by abnormal albumin) and phosphate amongst others to detect conditions associated with hypercalcemia, such as primary hyperparathyroidism or hypocalcemia and consequent secondary hyperparathyroidism causing bone loss [66]. Serum creatinine measurements and estimations of the glomerular filtration rate are also useful to detect renal failure which can affect bone health [66]. Although serum alkaline phosphatase (ALP) is a useful marker to detect certain disease conditions regarding bone, it is not sensitive enough to detect changes in bone remodelling [66].

Adequate supply of vitamin D is considered essential for bone health as it aids calcium absorption in order to maintain the calcium concentration in the physiologically-relevant range [67, 68]. Vitamin D levels are directly related to BMD irrespective of ethnicity and gender, maximum density achieved with levels of 40 ng/mL (99.8 nmol/L) or higher [69]. Since vitamin D is vital to the regulation of calcium and phosphorous metabolism, bone formation and mineralization, it is documented as one of the key factors associated with bone mass and therefore important in the management of osteoporosis [70]. Although the optimum level of serum 25(OH)D (the inactive form of vitamin D) in a healthy population is not uniformly accepted, a concentration of at least 30 ng/mL (75 nmol/L) is recognised as the endpoint (lowest) for skeletal health outcomes by most [70–72], whereas 20 up to 30 ng/mL (50–75 nmol/L) is considered acceptable and desirable [73].

Exposure of the skin to sunlight, the melanin concentration, clothing style when exposed to sunlight and the season all play important roles in vitamin D production [74, 75]. Age, BMI, vitamin D supplementation and physical activity also influence the concentration of serum 25(OH)D [72, 75]. Furthermore, serum 25(OH)D concentration also positively correlates with BMD and negatively with parathyroid hormone (PTH) [76–79], and it was determined that serum PTH tended to be higher in individuals with serum 25(OH)D between 50 and 75 nmol/L (20 up to 30 ng/mL), a concentration sufficient to prevent secondary hyperparathyroidism. PTH is primarily responsible for converting 25(OH)D in the kidney to the hormonal, active form of 1,25(OH)2D. However, many other tissues in the body are also able to instigate this conversion in order to act locally as a paracrine or autocrine hormone [80].

**Fig. 3:**
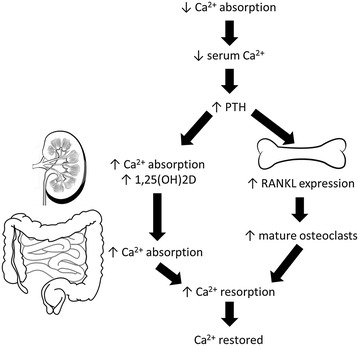
The restoration of suboptimal calcium levels

Directly linked to vitamin D’s role in calcium homeostasis, is the relationship between calcium and PTH (Fig. 3). When calcium absorption is sub-optimal, serum calcium levels decrease which in turn stimulates the production of PTH from the parathyroid glands [81]. This increases the reabsorption of calcium and enhances the expression of the receptor activator of nuclear factor kappa B (NFκB) ligand (RANKL) on osteoblasts to increase the production of mature osteoclasts which mobilizes calcium to bone, restoring serum calcium concentrations to normal [82]. PTH also mobilizes phosphorous from bone, as well as decreases kidney phosphorous reabsorption which causes phosphaturia [82]. Since PTH is regulated by calcium intake, it has been suggested that serum PTH measurements be taken if serum calcium is abnormal to determine the cause of abnormality [66].

Therefore, calcium and vitamin D deficiency are particularly important role players in bone health, because reduced calcium absorption increases PTH concentration and accelerates the rate of bone loss, which raises the number and activity of osteoclasts that release calcium from bone. Though this is an appropriate short-term homeostatic response, the long-term effects are detrimental to the skeleton because of the ongoing imbalance at the remodelling sites.

Additionally, locally produced growth factors such as insulin-like growth factor (IGF)-1 and -2, transforming growth factor (TGF)-β, interleukins (IL)s, prostaglandins, tumour necrosis factor (TNF)-α and osteoprotegrin ligand (OPG-L) have also been implicated as regulators of bone remodelling, together with the hormones oestrogen and androgens. These factors will be discussed at a later stage in this review.

### Clinical significance of BMD measurements

Bone status is largely absent in SA due to lack and cost of specific equipment for assessing BMD. A few studies suggested that BMD in Africa may be similar to northern world observations, however, the prevalence of fractures appears to be much lower [83–85].

With the use of alternative measuring techniques that are readily available for BMD, the cost will decrease; however, the precision of other bone density measuring techniques compared to DEXA is still not agreed upon. Nevertheless, the potential of diagnosing a person with the predisposition of developing osteoporosis, using easy to use equipment and markers associated with BMD is warranted as proper and earlier treatment may prevent or even delay the onset of osteoporosis. Confirmation however is still necessary with the use of a DEXA scan. HIV specifically has a negative impact on BMD and will be the focus of the next section.

## HIV

### HIV disease profile

With HIV infections, various complications arise which affect different systems of the body. These include cardiac, metabolic, endocrine, renal and inflammatory/immune systems to name but a few. Apart from these complications, changes in lipid and glucose metabolism, adipose tissue disorders and abnormal bone metabolism are also prevalent. One of the major focuses of research has been the association between HIV, HAART, metabolic disorders and vascular disease. However, the specific effects of HIV on the metabolic, endocrine and renal systems, as well as the bone, and the interactions between these systems have not been that extensively studied. Furthermore, although the use of HAART has improved the prognosis of HIV-infected patients dramatically, long-term HAART is associated with several cardio-metabolic, endocrine, renal and bone perturbations, which will be discussed in the next section.

#### HIV and the metabolic system

Insulin resistance, impaired glucose tolerance, impaired fasting glucose and diabetes mellitus (DM) are a spectrum of glucose metabolism disorders reported in persons with HIV infection both with and without HAART. In a HIV-positive population, approximately 25–35% of patients have impaired glucose tolerance, whereas 2–7% have DM [86, 87]. Genetic susceptibility and lifestyle seem to be some of the factors that most often cause variation in the prevalence of HIV from one population to another [87].

There is a strong association between insulin resistance and lipid accumulation in muscle and liver tissue in HIV-positive patients with lipodystrophy—increased VAT is associated with excess free fatty acids, increased intramyocellular fat content and reduced adiponectin [88]. Glucose uptake into abdominal subcutaneous adipose tissue (SAT) is increased in HIV-infected patients, whereas glucose uptake in VAT and muscle cells does not differ [88].

With specific reference to BMD, inconsistencies exist regarding the relationship between VAT and BMD. Whilst some studies suggest an inverse relationship [89, 90], a study by Yamaguchi et al. suggests a potential protective effect of VAT especially in patients with DM [91]. A study by Bredella et al. confirmed the findings of an inverse relationship between VAT and BMD in pre-menopausal obese women [92]. They further go on to suggest that this effect may at least by partially mediated by IGF-1, since IGF-1 and muscle mass are positive predictors of BMD. The growth hormone (GH)/IGF-1 axis is a major determinant of BMD [93, 94] and seems to be dysregulated in females specifically in proportion to the degree of VAT [95]. Other potential explanations could include the release of proinflammatory cytokines secreted by adipocytes, such as IL-6, TNF-α, and adipokines, such as E-selectin and adiponectin, stimulating osteoclast activity [90, 96, 97].

A previous study also found that lean body mass is an important predictor of BMD in premenopausal women, whereas fat mass correlated positively with BMD of the hip [98]. The adipokine, leptin, is responsible for regulating several hypothalamic pituitary adrenal (HPA) axes which are involved in mediating the connection between leptin and bone by means of direct and indirect mechanisms. Since the leptin receptor can be found in adult primary osteoblasts, it is suggested that leptin has a direct effect on bone growth through its inhibitory action against bone resorption (osteoblastic activity) [99]. It regulates osteocalcin which in turn regulates bone metabolism [100]. Furthermore, bone marrow adipocytes also secrete leptin which may mediate leptins’ local effect on bone. On the other hand, indirect mechanisms are more complex and involve cortisol, thyroid and parathyroid hormones, as well as growth hormones and IGF-1 [101].

#### HIV and the endocrine system

A wide spectrum of endocrine complications is associated with HIV infection and acquired immunodeficiency syndrome (AIDS). However, these disorders may also be as a consequence of systemic illness, opportunistic infections, body composition and/or HIV-related therapies [102]. The pituitary, thyroid, adrenal gland, gonadal, pancreas and bone are all involved.

Although HIV brings about secondary issues such as endocrine dysregulation, hormonal imbalances are not a common occurrence. HIV does however involve almost all of the hormonal systems and axes. One of the most common endocrine axes to be affected by HIV is the HPA axis [103]. During a condition such as HIV, viral proteins and cytokines activate or inhibit the hormonal system, further contributing to HPA dysregulation, a condition which is exacerbated with HAART [103]. The adrenal gland malfunctions as a result of adrenal insufficiency. Furthermore, due to a glucocorticoid resistant state, impaired adrenal and pituitary reserves cannot react to increased cortisol levels. Various different thyroid hormone disorders also occur which is associated with altered metabolism, poor oral intake and increased prevalence of weight loss and wasting syndrome [103].

Oestrogens and androgens have also been implicated to contribute to changes in BMD, not only during adulthood, but also during the growth of the skeleton. Oestrogen specifically has been shown to inhibit osteoclast function, and stimulate osteoblasts to produce growth factors and cytokines that mediate oestrogen’ action, some of which regulate the osteoblasts indirectly [104]. More importantly, oestrogen may play a role in determining the life span of bone cells by controlling the rate of apoptosis, thus decreasing the lifespan of osteoblasts and increasing the longevity of osteoclasts [104]. Two specific and complementing mechanisms have been implicated to explain the effect of oestrogen on bone. Firstly, oestrogen deficiency (most commonly as a result of menopause) causes bone loss through its activation of new bone remodelling sites and through exaggeration of the imbalance between bone formation and resorption [104]. Oestrogen deficiency stimulates osteoblast production of IL-1 and -6 and TNF-α and inhibits apoptosis, thus extending the life span of osteoclasts. Oestrogen deficiency decreases IL-1ra leading to enhanced osteoclast sensitivity to IL-1. These actions mainly occur via oestrogen receptor (ER)α, which then elicits a prominent effect on the regulation of bone turnover and the maintenance of bone mass [105]. Although osteoblast numbers are also elevated, the increase in bone formation is not sufficient to replace the bone matrix removed by osteoclasts, resulting in net bone loss.

One has to take into consideration that HIV-infected subjects already have low BMD, and even more so when treated with HAART. Given the vast proportion of women living with HIV, who are and will be transitioning through menopause, one also needs to consider the additional burden that low oestrogen levels will place on a BMD level that is already compromised by HIV and HAART. Statistics in the USA are largely alarming, as it is anticipated that by 2020, more than 50% of HIV infection will be in patients over the age of 50 years [106]. In women living with HIV, an intricate relationship between HIV and menopause appears to exist in that HIV may influence the natural history, experience, and complications of menopause, while menopause itself could potentially influence the course of HIV infection [107]. This relationship between HIV and menopause further complicates the management of HIV infection in women as they age and challenges clinicians, since lymphocyte subsets, including CD4 counts have been found to decrease with increasing age in non-HIV-infected adults [108]. Considering that older individuals are usually diagnosed later with a more advanced stage of HIV, CD4 counts will be even lower. Therefore, the combination of all of these factors predisposes women to an earlier and even more severe bone loss compared to those individuals without HIV.

In males, testosterone affects BMD through its direct effect on the androgen receptors in trabecular and cortical bone [109, 110]. Testosterone’s effect is more complex than that of oestrogen as it is converted to estradiol in adipose tissue or bone, with subsequent stimulation of the oestrogen receptors [110–112]. When androgen levels are low, it is associated with bone loss and increased bone remodelling in men [113, 114]; however, a reduction in oestrogen seems to play a key role in the age-related decrease of BMD [115, 116]. With advancing age, testosterone is also considered the major source of circulating estradiol in women (menopause), and testosterone can regulate local production of cytokines and growth factors in bone, including IL-6, IL-1β, TGFβ and IGFs [109]. The anabolic effects of testosterone on muscle mass and strength can also indirectly affect bone mass.

Data on the effect of low testosterone levels on BMD are not in agreement. One specific study suggests that serum total estradiol, but not testosterone is associated with reduced BMD in HIV-infected men [117]. This study showed that among men with low testosterone, it is only those men that develop an oestrogen deficiency that will have lower BMD, a result which was also evident in other studies [118, 119], although some studies found the opposite to be true [120, 121].

It is therefore evident that older age, and therefore the decrease in sex hormones, impacts BMD even more negatively when associated with HIV and HAART treatment.

#### HIV and the renal system

Patients with HIV usually show signs of proteinuria which progress rapidly to end-stage renal disease, commonly referred to as HIV-associated nephropathy (HIVAN) [122]. The nucleoside reverse transcriptase inhibitor (NRTI), tenofovir (TDF), has been linked to proximal renal dysfunction (PRTD), which can result in excessive renal phosphate, uric acid and bicarbonate loss. Furthermore, it can also result in proteinuria and glucosuria especially in patients with pre-existing nephropathy [123]. A study by Fux et al. showed that PRTD may promote BMD loss through renal phosphate wasting [124]. Studies originally reported on nephrotoxic effects as a result of TDF, but the incidence of adverse renal effects were low in initial randomized trials [125, 126]. A small but significant loss in renal function was observed in patients on TDF-containing regimens compared to abacavir (ABC)-containing regimens [127]; however, studies are not conclusive regarding renal endpoints and more patients developed clinical renal impairment [128, 129]. Additionally, although both the STEAL and ASSERT study indicated no difference in estimated glomerular filtration rate (eGFR) between patients [130, 131], increases in markers of proximal tubule dysfunction were evident as a result of TDF treatment in the ASSERT study [130]. This is comparable to results obtained by Rasmussen et al. In this particular study, although no difference in renal function was evident when plasma cystatin C (cysC) and estimated creatine clearance (CrCl) were measured (indicative of stable GFR), a minor increase in urinary neutrophil gelatinase-associated lipocalin (NGAL)/creatinine ratio (as a marker of renal dysfunction) was observed in the TDF-containing regimen compared to the ABC-containing regimen [132]. CrCl is however dependent on factors other than plasma creatinine and GFR, such as changes in muscle mass and tubular secretion of creatinine.

A different formulation of TDF was approved by the FDA in order to try and overcome the side effects associated with TDF. Patients that switched to tenofovir alafenamide (TAF) seemed to be more likely to keep their viral load low, whilst kidney function and bone density was improved [133, 134]. Even after 2–3 years, TAF was better at suppressing viral load and safer for the bones and kidneys [135, 136]. A similar study by Brown et al., also showed that participants who switched from TDF to TAF showed improved bone health, including a reduction in osteoporosis [137].

#### HIV and bone mineral density

A reduction in BMD is a common complication of HIV and its treatment, with low BMD, osteopenia and osteoporosis more commonly associated with both male and female HIV-infected subjects [138, 139], compared to non-infected subjects [140, 141].

The rates of osteopenia and osteoporosis were found to be as high as 67 and 15% respectively, whilst the magnitude of BMD reduction was 6.4-fold higher, and that of osteoporosis 3.7-fold higher in HIV-positive patients, as reported in a meta-analysis [142]. Cazavane et al. indicated that more than half the HIV-infected patients had osteopenia, and approximately a third had osteoporosis [138], results which were verified by a study from McComsey et al. [143]. Triant et al. also reported a higher prevalence of fracture risk in HIV-positive patients compared to HIV-negative patients [144]. A more recent study reported the rates for osteopenia and osteoporosis to be 47.5 and 23% respectively, whilst BMD decreased in about 28% of subjects in a follow-up study of 2.5 years [145]. Sharma et al. found similar results, where rates of 42 and 12% were reported for osteopenia and osteoporosis respectively—the degree of osteopenia was three times higher in the HIV-infected subjects with 12% of patients progressing to osteoporosis [146]. These studies highlighted the existence of high prevalence of osteopenia and osteoporosis in the HIV-positive population.

HIV-positive patients have reduced bone size, mass and strength due to altered metabolism and the infection of bone cells [147]. It has been suggested by Duvivier et al. and Borderi et al. that HIV infection of osteoblasts may be related to a negative balance of bone remodelling [148, 149]. Since osteopenia and osteoporosis is highly prevalent in HAART-naïve patients, it suggests that if viremia is not controlled properly, it might impact BMD via its effects on persistent systemic inflammation and bone remodelling. Not only do the HIV proteins increase osteoclast activity, but they promote osteoblast apoptosis, thereby decreasing bone formation [150].

Under normal conditions, both vitamin D and phosphate metabolism are important for skeletal integrity; however, in HIV, vitamin D and phosphate contributes to low BMD due to impaired mineralization [148]. Approximately 60–75% of patients in a HIV-infected population have low vitamin D levels [151]. Furthermore, hypogonadism may also play a role [152], and lipoatrophy seems to mediate bone loss through adipocyte hormone signalling [89].

Since persistent systemic inflammation can impact bone density, the involvement of various cytokines involved in this process are of great importance. T-cells (specifically targeted by HIV infection), B-cells and monocytes are responsible for the production and regulation of key osteoclastogenic cytokines such as RANKL and OPG [153]. It is speculated that HIV infection results in the alteration of B cell function and subsequently a switch from bone sparing OPG production to the production of bone destroying RANKL, thus leading to increased osteclastogenesis [154]. This data is consistent with the fact that B-cells are mainly regulated by interactions with T-cells, as well as the fact that severe perturbations of the B-cell linage are mediated by HIV infection (through direct effects of viral infection and/or indirectly though disruption of co-stimulatory signals from T-cells and other disrupted immune components) [155, 156]. Along with upregulation of RANKL by HIV gp120 protein, there is further activation of RANKL by TNF-α that is represented in higher numbers in advanced HIV disease [157].

A dramatic increase in the number of osteoclast precursors (defined as monocytes expressing the RANKL receptor, RANK) were also evident, whilst OPG expression was significantly diminished [156]. Data from animal studies showed that HIV infection results in alterations in the immuno-skeletal interface favourable to accelerated bone resorption and loss of bone mass. Low serum OPG concentrations have been reported in HIV/AIDS patients [158], verifying some of the data seen from animal studies. Furthermore, peripheral blood T-cells have been implicated as a potential source of OPG [158]. Other studies have shown that HIV-infected women have higher levels of both RANKL and OPG than their HIV-negative counterparts, suggesting increased overall bone turnover in HIV-infected patients [159], while HAART initiation has been associated with increases in both, again suggesting increased bone turnover [160, 161].

One of the most recent studies performed were able to show that the combination of all B cells were responsible for the increase in RANKL, whilst the decrease in OPG was due to changes in the distribution of B cell subsets [162]. Therefore it is crucial to distinguish between the different B cells and to include naïve and resting memory B cells (decreased number with high expression of OPG), and tissue-like B cells (increased number with low expression of OPG). When the relationship between these markers and BMD in HIV-positive participants was investigated, RANKL/OPG correlated significantly with BMD and T- and/or Z-scores in the femur neck and hip region, which was not evident in the HIV-negative participants. The data therefore supports the concept of B cell alterations in RANKL and OPG production which may contribute to the decline in BMD in the context of HIV. Interestingly, although a decrease in OPG expression in the B cells was observed overall, serum OPG levels were elevated whilst RANKL was decreased, which was also shown in other studies [163, 164]. However, whereas some of the studies showed a decrease in RANKL, the study by Gibellini et al. indicated an increase in RANKL suggesting that relying only on circulating markers is not a true reflection of what happens in bone.

Interferon (IFN-γ) has also been shown to interfere with the RANKL receptor through its action on the adapter protein, (tumour necrosis factor receptor-associated factor-6; TRAF-6) that links RANK to its downstream transcription factors, ultimately inhibiting osteoclast formation [165, 166]. These alterations in the immune-skeletal interface may therefore account for much of the loss of BMD observed in HIV patients.

Even though there are growing concerns regarding osteoporosis, the impact of HIV and its treatment on bones in resource-limited countries is poorly documented.

### HAART and bone mineral density

Various studies have reported on low BMD in HIV-infected adults; however, the prevalence of osteoporosis seems to be approximately three times higher in HIV-infected patients receiving HAART treatment than uninfected patients [142]. Although studies reported a 2–6% decrease in BMD (within the first 2 years) with HAART treatment [149, 167], this decline in BMD only pertains to patients starting a new HAART regimen (within 24–48 weeks) [149, 168], and not continuing on a currently established regimen. This decrease seems to be similar to that observed within the first 2 years of menopause [169].

From the literature it is evident that the specific type of treatment can have a significant effect on bone loss. The use of NRTIs has been associated with low BMD [160, 168, 170]. TDF-based treatment patients had a much lower BMD after 96 weeks of treatment compared to other regimens such as lamivudine (3TC) and emtricitabine (FTC) patients, although no higher incidence of fracture were evident [160, 168, 170]. Similar results were obtained in a study using ABC as alternative [160].

Results from the study of Cotter et al. showed no significant alteration in phosphate metabolism that often accompanies bone loss [171], although some studies have shown TDF is able to decrease BMD through persistent urinary loss of phosphates [170, 172]. Instead, higher serum phosphorus among patients with low bone density was evident, although it is not exactly clear why. Furthermore, time on TDF showed a trend to correlate with low BMD Z-scores, which is in direct contrast to other studies which have failed to show any correlation between TDF and low bone mass, even after long-term exposure to the drug [173, 174]. NRTIs can further reduce BMD by elevating lactic acidemia, a mechanism related to calcium hydroxyapatite loss, especially in the trabecular bone, due to the lability of calcium storage [175]. Several studies have directly highlighted HIV factors associated with low BMD: duration of infection, HIV viral burden, and a more advanced HIV disease [171].

Studies regarding PIs remain unclear due to conflicting results. PI use has been shown to result in increased bone turnover, accelerated bone loss, and a higher prevalence of reduced BMD [142, 149, 170, 176], whilst other studies failed to show such a difference [167, 177–179]. A recent study conducted in Asia by Kinai et al. indicated that PI use, but not specific type of PI, was the most significant cause of low BMD at both spine and femoral neck [180]. This finding is consistent with an in vitro study evaluating the effect of different PIs on osteoblast activity [181]. Kinai et al. also observed a large difference in BMD between PI-discontinued and continued patients—PI discontinued patients displayed BMD levels in the lumber spine and not femoral neck [180], consistent with other studies [149, 170]. This is likely due to the fact that the femur contains cortical substance with few osteoclasts, whilst the vertebrae contain osteoclast-rich trabecular substance. Interestingly, discontinuation of ritonavir (RTV), an osteoclast-activating agent, resulted in slower decrease in BMD in vertebrae compared to the femoral neck [180]. Furthermore, RTV was able to cause bone mineral loss in a time dependent manner irrespective of dosage [180]. RTV was also shown to promote the proliferation and activation of osteoclasts in both in vitro [182, 183] and ex vivo studies [184], causing increased bone absorption.

In a study by Duvivier et al., a four percent decline in BMD were evident after 48 weeks on HAART in the lumbar spine and a three percent decline in hip BMD [149]. Although BMD loss occurred with PIs, NNRTIs alone, as well as a combination of PIs and NNRTIs, could not worsen the BMD loss in the spine [149]. In contrast, a significant loss in BMD (5–6%) at the femoral neck by week 48 were evident irrespective of NRTI or PIs [185].

Specific BMD measurements with DEXA showed that immune suppression before starting HIV therapy was a risk factor for loss of BMD during treatment. However, there was no evidence that the extent of immune reconstitution was associated with BMD change after controlling for baseline CD4^+^ count even with HAART initiation—loss of bone continues after HIV therapy is initiated (up to 2 years) [186]. Data therefore suggests that low pre-treatment CD4 count, but not early change with HAART was a strong and independent risk factor for bone loss after HAART initiation—providing further evidence for the benefits of early initiation of HAART [186].

Although HAART may be associated with the occurrence of fractures, none of the studies thus far were able to show any changes in fracture rates. This is possibly due to the limited number of cohort studies exploring this phenomenon, as well as a too short follow-up period.

### The mechanisms of action of HAART-induced bone loss

The specific mechanisms by which HAART induces bone loss remain debatable, but it has been thought to be either due to a direct effect on bone cells (most likely), realignment of HIV-associated pathologies resulting from disease reversal, or a combination of these factors. In vitro and in vivo studies involving animal models have shown that HAART do act on osteoclasts and osteoblasts; however, these effects could not be verified in humans due to the different effects the different HAART classes have on bone. One such example was found in a study by Wang et al. in which the PI, RTV, suppressed osteoclastogenesis and osteoclast function in vitro and in vivo by impairing RANKL-induced signalling, but the PI indinavir (IDV) had no effect on osteoclastogenesis [187]. The PI fosamprenavir (FPV) significantly increased OPG and decreased RANKL production in vitro, which could suggest protection of bone mass if confirmed in vivo. In contrast, other PIs including atazanavir (ATV), saquinavir (SQV), and IDV failed to impact the OPG/RANKL ratio [181]. Furthermore, preliminary studies in vitro have found no effect of several NRTIs on osteoclastogenesis, but NRTIs were found to suppress osteoblast activity instead [188].

### Confounding factors and discrepancies in results

Many confounding factors are assumed to play a role in the pathogenesis of decreased bone mass in HIV-infected patients [189]. Fat-free mass, one of the most important components of body composition was shown to be one of the problems [190]; however, according to Reid et al. [191], low weight appears to be the major component in healthy individuals, which has also been verified in a HIV-positive population [147]. Results from a more recent study are in accordance with the important role of body composition and have indicated that weight, BMI, and body fat were all associated with BMD, especially in females [192], which also highlights the importance of nutrition on BMD, especially because low calcium and vitamin D intake reduces BMD [193]. A study in Brazil indicated that HIV-positive populations have calcium and vitamin D deficiencies, even though their dietary intake is similar to that described in other young Brazilian populations [194, 195]. Limitations in studies regarding nutrition and BMD are often due to the fact that participants need to recall what they have consumed and errors might slip in as it is self-reported. Low BMD have been linked to low body weight, testosterone or oestrogen deficiency, glucocorticoids, malabsorption, tobacco use, alcohol and opiate abuse, nadir CD4 cell count, duration of HIV infection, lipodystrophy, insulin resistance and hyperlactatemia, all of which could contribute to the discrepancies in results [196].

## Conclusions

Although bone loss is a common complication of HIV, HAART initiation further worsens, rather than ameliorates, bone loss. This effect of HAART on BMD is rather alarming in a population whose skeletal integrity is already seriously compromised due to HIV infection. The interaction between bone and the endocrine, metabolic and renal system in this population complicates bone dynamics even further. Opinions still differ widely among investigators as to the direct effects of HAART or their components on bone cells, or their mechanisms of action on the skeleton. Consequently, it is rather thought that all HAART formulations may be inherently detrimental to the skeleton as bone loss appears to be a general phenomenon observed following HAART initiation, regardless of the regimen type, although PIs and specifically TDF may be the main causes.

Various studies found that continuous exposure to HAART results in a further decline in BMD compared with intermittent HAART exposure. Although most of the important questions regarding HAART-related bone loss are now answered, such as: (1) Are certain HAART regimens more detrimental than others? (2) How soon after HAART initiation does bone loss begin? and (3) Does it occur in a specific timeframe or is it a long-term effect? the fact still remains that HAART treatment has a negative influence on BMD.

The NRTI, TDF has been associated with bone loss in various studies compared to other regimens. PIs on the other hand have also been associated with skeletal deterioration in most studies. While the degree of bone loss may vary somewhat between regimens, almost all HAART regimens resulted in a significant loss of BMD between 2 and 6% over time. The biggest loss following HAART initiation appears to occur within 24–48 weeks. Furthermore, once lost, BMD never recovers to pre-treatment baseline levels, let alone to normal BMD levels characteristic of uninfected individuals of similar age. Therefore, it is imperative that BMD be monitored and treatment started if a patient is diagnosed with low BMD.

Determining BMD in a specific population also appears to be somewhat of a concern, since the gold standard method to determine BMD, the DEXA, is not readily available in all clinics and areas. The ability to use other assessment tools such as QUS in resource limited settings will have to be investigated, but validation of the method needs to first take place before being able to use it as a diagnostic and research tool for a specific population.

*Take home message to clinicians* The primary goal for any clinician in treating people with osteoporosis is preventing fractures. However, when the patient is faced with HIV or even the additional burden of HAART treatment, the strategies should probably have to be altered. With respect to BMD management, those individuals with HIV should undergo the same treatment process as HIV-negative people, and should also include lifestyle modifications and pharmacological interventions. This will be of great benefit not only when BMD is low, but will also benefit those individuals with HIV. One should always bear in mind that certain pharmacological interventions for other conditions, may have interactions with HAART medication in HIV subjects and might not be safe to use. Some of the medications taken for osteoporosis have also not been tested in combination with the various HAART treatment options. These patients should therefore be carefully monitored for a couple of months to establish whether any side effects arose as a consequence of treatment. For the most part, the benefits of HAART with respect to virologic control are considered to outweigh the risk of potentially exacerbating bone loss. However, in those patients who are at high risk of a fracture, clinicians may want to consider avoiding TDF or PIs if alternatives are available. When also faced with the additional burden of menopause, one has to take into consideration that oestrogen is also now low, and that if oestrogen is given additionally, it might have an effect or even interfere with the other medications already prescribed.

Usage of FRAX is recommended, and secondary causes of osteoporosis other than HIV or menopause should be evaluated. Patients at high risk for fractures should be encouraged to make lifestyle changes, to avoid tobacco and alcohol, and to prevent falls. Once osteoporosis has been diagnosed and therapy started, DEXA should be repeated every 1–2 years as follow-up.
